# Impact of breath-hold level on positional error aligned by stent/Lipiodol in Hepatobiliary radiotherapy with breath-hold respiratory control

**DOI:** 10.1186/s12885-020-07082-y

**Published:** 2020-07-01

**Authors:** Tzu-Jie Huang, Yun Tien, Jian-Kuen Wu, Wen-Tao Huang, Jason Chia-Hsien Cheng

**Affiliations:** 1 Division of Radiation Oncology, Department of Oncology, National Taiwan University Hospital, National Taiwan University College of Medicine, No. 7, Chung-Shan South Rd, Taipei, 10002 Taiwan; 2 Department of Medical Imaging and Radiological Technology, Yuanpei University of Medical Technology, 306 Yuanpei Street, Hsinchu, 30015 Taiwan; 3 Taoyuan Psychiatric Center, Ministry of Health and Welfare, No.71, Longshou St., Taoyuan, 33058 Taiwan; 4 School of Medicine, College of Medicine, Fu Jen Catholic University, New Taipei, Taiwan; 5 Graduate Institutes of Oncology, National Taiwan University Hospital, National Taiwan University College of Medicine, Taipei, Taiwan; 6 Graduate Institutes of Clinical Medicine, National Taiwan University College of Medicine, Taipei, Taiwan

**Keywords:** Radiotherapy, Breath holding, Hepatocellular carcinoma, Patient positioning, Radiotherapy planning, Computer-assisted/methods

## Abstract

**Background:**

Respiratory motion management with breath hold for patients with hepatobiliary cancers remain a challenge in the precise positioning for radiotherapy. We compared different image-guided alignment markers for estimating positional errors, and investigated the factors associated with positional errors under breath-hold control.

**Methods:**

Spirometric motion management system (SDX) for breath holds was used in 44 patients with hepatobiliary tumor. Among them, 28 patients had a stent or embolized materials (lipiodol) as alignment markers. Cone-beam computed tomography (CBCT) and kV-orthogonal images were compared for accuracy between different alignment references. Breath-hold level (BHL) was practiced, and BHL variation (ΔBHL) was defined as the standard deviation in differences between actual BHLs and baseline BHL. Mean BHL, ΔBHL, and body-related factors were analyzed for the association with positional errors.

**Results:**

Using the reference CBCT, the correlations of positional errors were significantly higher in those with stent/lipiodol than when the vertebral bone was used for alignment in three dimensions. Patients with mean BHL > 1.4 L were significantly taller (167.6 cm vs. 161.6 cm, *p* = 0.03) and heavier (67.1 kg vs. 57.4 kg, *p* = 0.02), and had different positional error in the craniocaudal direction (− 0.26 cm [caudally] vs. + 0.09 cm [cranially], *p* = 0.01) than those with mean BHL < 1.4 L. Positional errors were similar for patients with ΔBHL< 0.03 L and > 0.03 L.

**Conclusion:**

Under rigorous breath-hold respiratory control, BHL correlated with body weight and height. With more accurate alignment reference by stent/lipiodol, actual BHL but not breath-hold variation was associated with craniocaudal positional errors.

## Introduction

Hepatocellular carcinoma is a common gastrointestinal cancer with no obvious symptoms at early stage or diagnosis. In recent years, radiotherapy has become a non-invasive treatment option. Many studies indicate that radiotherapy improves the local control rate and median survival of patients with liver cancer [1–3]. With the recent development of linear accelerators and radiotherapy technology such as multi-leaf collimators, flattening filter-free mode, image-guided radiotherapy (IGRT), and stereotactic body radiotherapy (SBRT), higher doses can be delivered to tumors for better biological effect because the treatment plan can be more complex and the dose gradient can be steeper [4, 5]. However, the liver is located near the diaphragm, creating a challenge to measure the tumor motion and deformation caused by respiratory movement. According to the American Association of Physicists in Medicine (AAPM) Task Group Report no. 76, motion management strategies should be used during radiotherapy in patients whose breathing motion exceeds 5 mm [6]. Deep inspiration breath-hold (DIBH) is one method which reduces the margin of planning target volume (PTV) and provides for accurate dose delivery [7]. Combining DIBH with IGRT in radiotherapy can enhance positioning reproducibility and facilitate dose escalation [8–11].

One spirometric motion management system, the so-called SpiroDynr’X system (SDX™ system), is a computer-controlled device that assists in voluntary breath hold. The system includes a very sensitive spirometer to quantify inspiration volume and establishes patient feedback by using video goggles, similar to virtual reality goggles. The patient can inhale to reach the defined target zone, then hold the breath while using visual data for reinforcement [12]. The preset breath-hold range of the SDX™ system can improve the reproducibility of the predetermined phase of the breathing cycle [13]. Thus, the use of the SDX™ system has been one of the breath-hold systems integrated into radiotherapy treatment for hepatobiliary cancer.

Of note, the changes in the inter-fraction liver position relative to vertebral bodies were significantly larger than in the intra-fraction liver position reported in previous studies [14]. A two-dimensional, offline imaging technique has been used to measure the motion of the liver tumor with other radiopaque markers used to correct the systematic error [15]. However, the random error generated in PTV with two-dimensional offline images guided by vertebral bodies led to geometry uncertainty and increased the radiation dose in the surrounding critical normal tissue [16].

The purpose of this study is to investigate the association of the breath-hold level (BHL), the variation in BHL, and the body-related factors with the positional errors in patients undergoing radiotherapy under a rigorous breath-hold control with SDX.

## Methods

### Patients

**Fig. 1:**
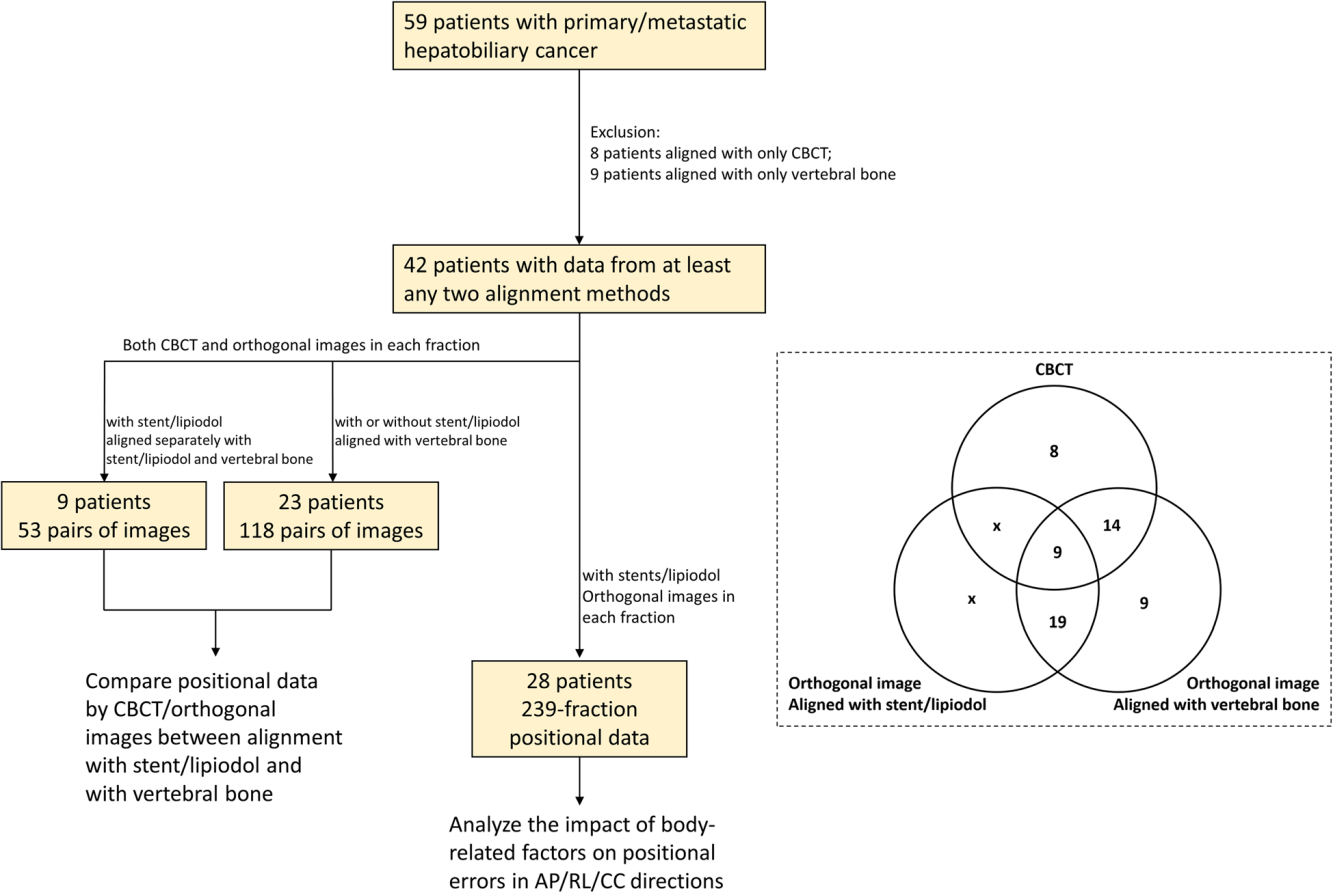
Flow chart of the recruited patients

We reviewed 59 patients (48 males and 11 females) who had primary or metastatic hepatobiliary cancer (liver, bile duct, and gallbladder) and underwent radiotherapy using the SDX system with normal lung function from May, 2014 to March, 2018. Among these 59 patients, we initially excluded 17 patients with only the data either from CBCT alignment or from vertebral alignment. The remaining 42 patients were eligible for the following two analyses. Twenty-three patients were analyzed to compare the correlation between CBCT and two alignment methods (vertebra and stent) on orthogonal images. Twenty-eight patients with either stent or embolized materials (lipiodol) were eligible for the analysis of body related factors. Flow chart of the recruited patients is shown in Fig. 1. Patient characteristics are listed in Table 1.

**Table 1:** Patient characteristics

Characteristics	Number	Percent
Gender		
Male	22	78.6
Female	6	21.4
Age		
Median	63.5	
Range	38–78	
Height (cm)		
Median	166	
Range	147–176	
Weight (kg)		
Median	64.9	
Range	40.2–81.7	
BMI		
Median	22.81	
Range	16.40–30.07	
Disease		
HCC	15	53.5
Liver metastasis	4	14.3
Pancreas cancer	3	10.7
Cholangiocarcinoma	4	14.3
Gallbladder cancer	2	7.2

### SDX system

The SDX system (SpiroDynr’X system®, Muret, France) was used in simulation and radiotherapy for patients with computer-controlled voluntary breath hold. The system includes the SDX module, video goggles, utility module, calibration syringe, laptop, and SDX software. The SDX module is comprised of a sensor connected to a mouthpiece and spirometer. Patient feedback can be set by using nose clips to force breathing from the mouth, and video goggles allow the patient to watch their own spirometry pattern to improve breath holding.

### Simulation and preparation

All patients were immobilized with vacuum cushions, and underwent computed tomography (CT) simulation using a Philips Brilliance Big Bore CT (Philips, Eindhoven, Netherlands) for treatment planning. When the CT images were acquired, patients were asked to inhale to reach the predefined range and then hold the breath. The target volume and organs at risk were contoured and planned using the Eclipse™ (V13.0, Varian Medical Systems Inc., Palo Alto, CA, USA) treatment planning system.

The BHLs of deep inspiration were practiced and determined in simulation. When patients used the SDX system for the first time, they breathed freely through the spirometer until being instructed to take a full inspiration in order to determine the inspiratory capacity; they did these three times to assure the reproducibility of breathing patterns. The BHL was defined as 85% of the maximum inspiratory capacity to ensure the patient’s tolerance to complete multiple breathing cycles during fractionated radiotherapy [17, 18]. Inspiration zone (breath-hold range) was defined as the BHL + 0.1 L (Supplementary Fig. 1A).

### Radiotherapy with image guidance

**Fig. 2:**
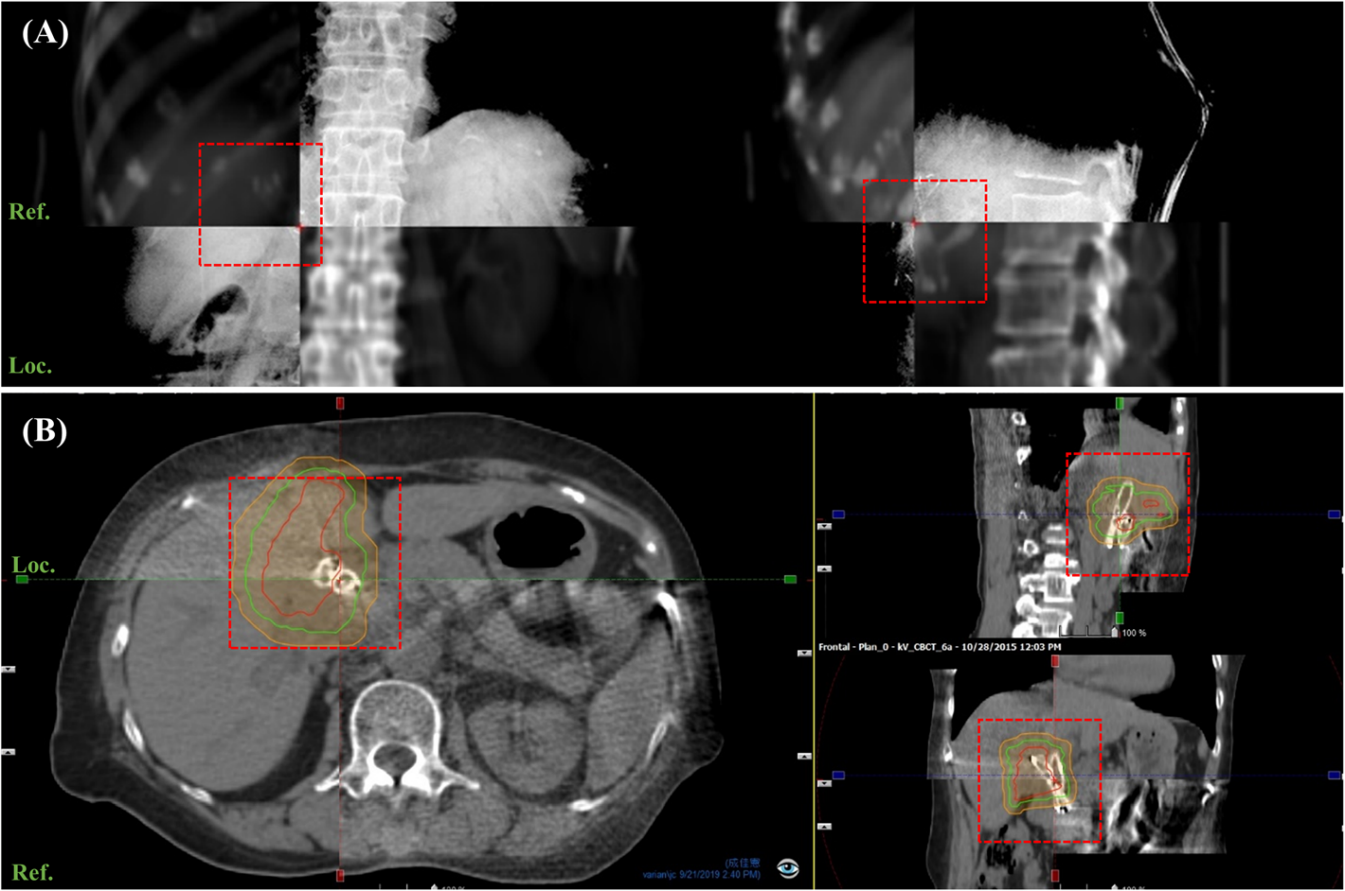
**a** Anterior-posterior and lateral kV-orthogonal images and (**b**) cone-beam computer tomography (CBCT) with 360-degree acquisition by Varian’s On-Board Imager® system were used to align (crosshair) target and liver with stent/lipiodol on simulation CT

The linear accelerator used for radiotherapy was the TrueBeam system (Varian Medical System Inc., Palo Alto, CA, USA), with 6 MV or 10 MV photons. The kV-orthogonal images (75 kV, 200 mA, 25 ms and 95 kV, 200 mA, 200 ms) or cone-beam (CB) CT (125 kV and 264 mAs) were taken before each treatment using Varian’s On-Board Imager® (OBI) system to confirm the accuracy of position, and the treatment couch was immediately adjusted to correct for the positional errors (Fig. 2). With the longer time required to take CBCT, some patients were not able to hold their breaths for acquiring CBCT. In comparison, kV-orthogonal images, which took shorter acquisition time, were technically applicable and more frequently used in our patients. Generally, the kV-orthogonal images were more frequently obtained than CBCT for the best acquisition in a single breath hold. CBCT was needed when the alignment of the treated targets required the structural information inside the liver, especially in patient with no placement of fiducial markers. For the treatment session, the breath-hold range was displayed on the SDX module and patients started taking a breath to reach the BHL (Supplementary Fig. 1B). Patients needed to maintain breath-holds for at least 25 s with the same inspiratory volume every time, for radiation dose delivery and image acquisition.

### Analysis of the accuracy of different image-guided alignment markers

**Fig. 3:**
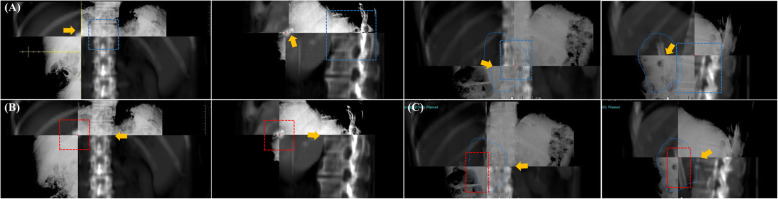
Alignment strategies with different markers on paired kV-orthogonal images with crosshair were based on (**a**) vertebral bony structure (upper panels), (**b**) lipiodol, and (**c**) stent (lower panels)

In each fraction of treatment, acquired CBCT or orthogonal kV images were compared with the planning images for the alignment and the inter-fractional positional errors by a qualified radiation oncologist. The inter-fractional positional errors were recorded in the anterior-posterior (AP), cranial-caudal (CC), and right-left (RL) directions. The shifts derived from CBCT alignment were used as baseline, and Pearson’s correlation coefficient was calculated to compare the accuracy of using different alignment markers on kV-orthogonal images (Fig. 3).

### Statistical analysis

This is a retrospective analysis of a patient cohort for DIBH in hepatobiliary radiotherapy. Each breath-hold value of patients during their treatment was collected. The BHL variation (ΔBHL) was defined as the standard deviation in difference between each breath-hold value and the baseline BHL. Mean BHL was defined as the average of each patient’s BHL during treatment. A total of 239 kV-orthogonal images by OBI system were analyzed for positional errors based on the stent/lipiodol position close to the tumor in the AP, CC, and RL directions. Patients were divided into two groups by the cutoff value close to mean BHL or ΔBHL to compare the position shifts. Body weight and height of patients were measured on the simulation day. IBM SPSS Statistics version 22.0 software (IBM Corp., Armonk, NY) was used for Pearson correlation analysis. Data were presented as the mean ± standard deviation for the indicated metrics. Differences between pairs of physique group were tested using the Student’s t-test, and a *p* value less than 0.05 was considered statistically significant.

## Results

**Fig. 4:**
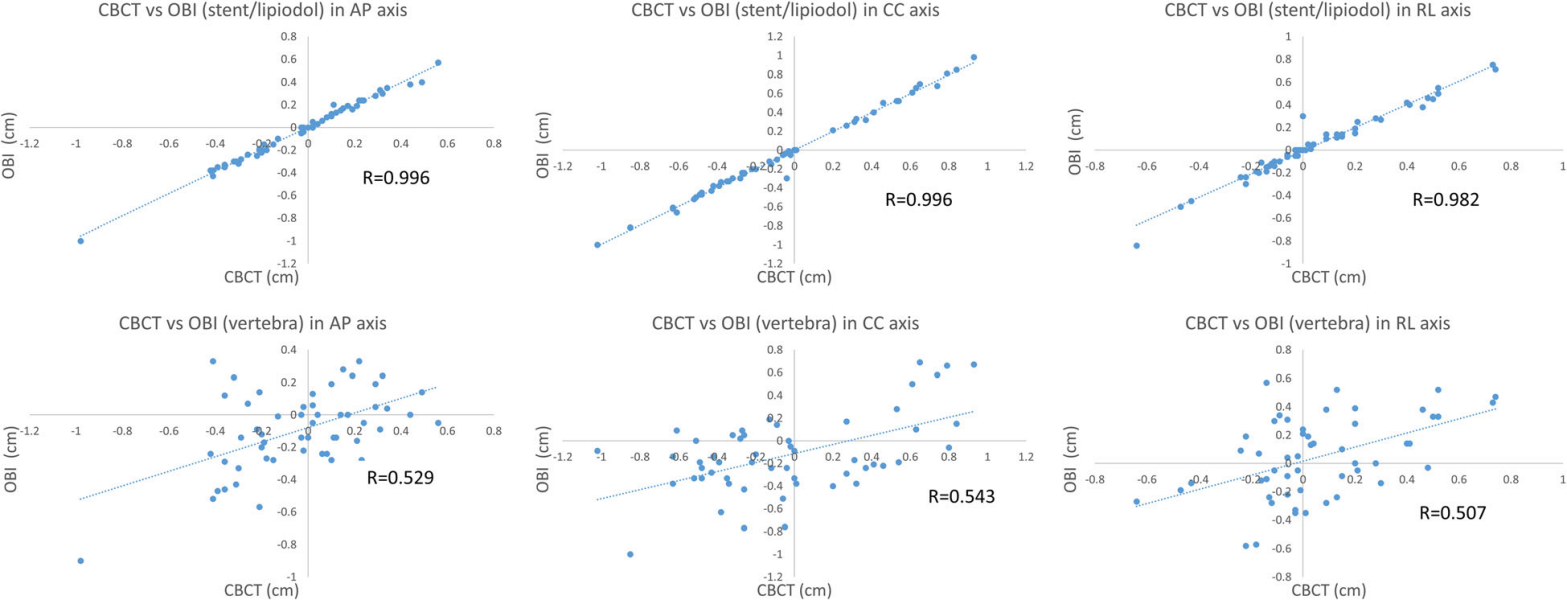
Significantly higher correlation of detected positional errors in anterior-posterior (AP), cranial-caudal (CC), and right-left (RL) directions between cone beam computed tomography and kV-orthogonal images by Varian’s On-Board Imager® (OBI) system when aligned with stents or embolized materials than with vertebral bony structure

Among 42 patients included in this study, 118 pairs of images from 23 patients (without stent/alignment) with both CBCT and kV-orthogonal images positioned with vertebral bones and 53 pairs of images from 9 patients (with stent/alignment) with CBCT and kV-orthogonal images aligned separately with vertebral bodies and with stent/lipiodol were analyzed. Pearson’s correlation coefficient was calculated to compare the accuracy of the two alignment methods on kV-orthogonal images. As shown in Fig. 4, correlation was significantly better with stent/lipiodol than with vertebral bone in the AP (*r =* 0.996, *p* < 0.001 vs. *r =* 0.529, *p <* 0.001), CC (*r =* 0.996, *p <* 0.001 vs. *r =* 0.543, *p <* 0.001), and RL axis (*r =* 0.982, *p <* 0.001 vs. *r =* 0.507, *p <* 0.001).

The 28 patients using the SDX system maintained a median breath-hold interval of 30 s (range: 25–40 s). The average BHL was 1.41 L (range: 0.76–2.08 L). ΔBHL ranged from 0.011 L to 0.041 L, with a median ΔBHL of 0.031 L. The positional errors in the AP, CC, and RL directions were − 0.05 ± 0.25 cm, − 0.09 ± 0.37 cm and 0.04 ± 0.24 cm, respectively.

**Fig. 5:**
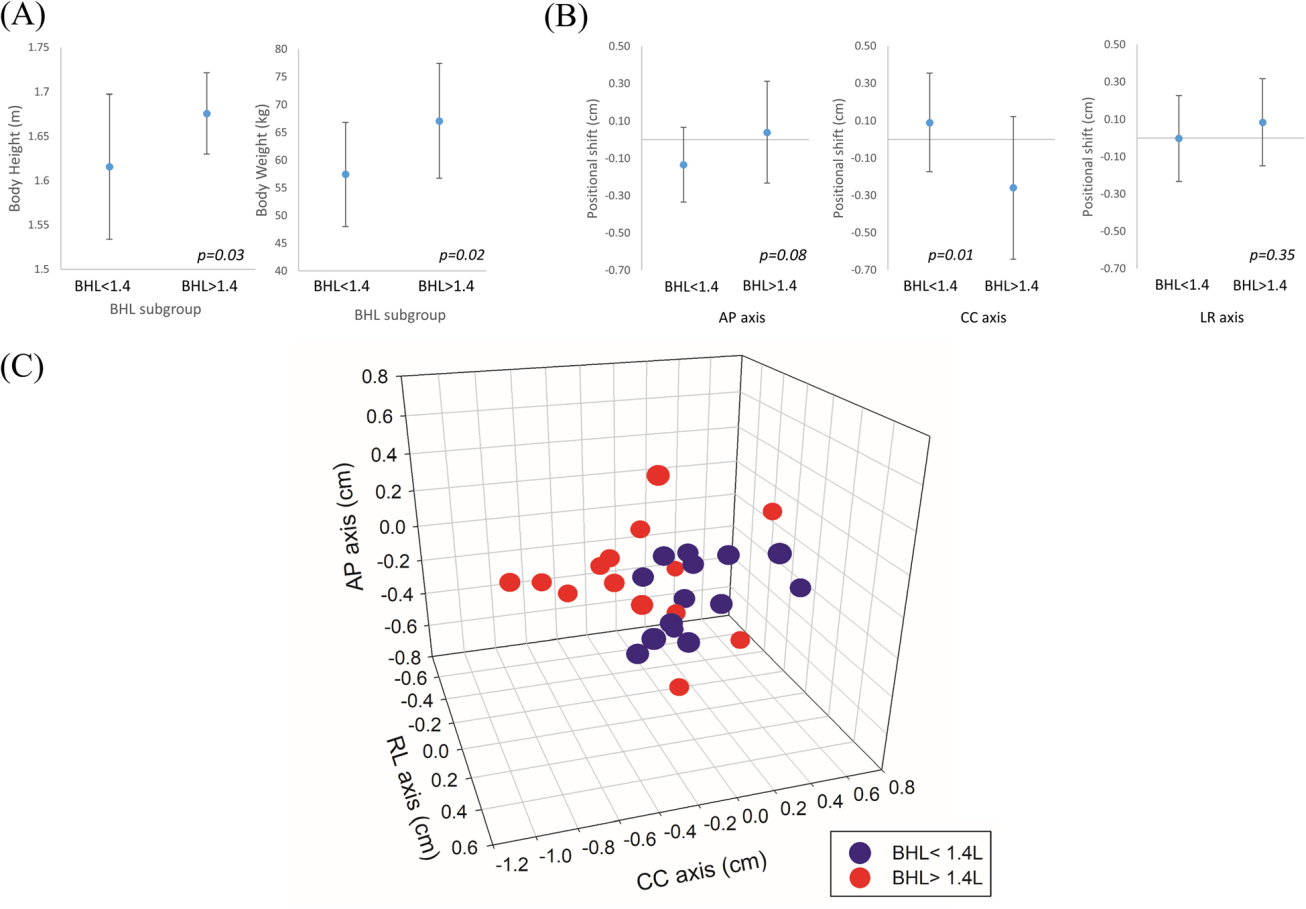
Differences in patients’ (**a**) body weight, height, (**b**) positional errors in anterior-posterior (AP), cranial-caudal (CC), and right-left (RL) directions between the subgroups of different mean breath-hold level (BHL). **c** The distribution of positional shift of each patient in all axes

Patients with mean BHL > 1.4 L were significantly taller (167.6 cm vs. 161.6 cm, *p* = 0.03) and heavier (67.1 kg vs. 57.4 kg, *p* = 0.02) than those with BHL < 1.4 L (Fig. 5a). In addition, significantly larger positional errors in the CC direction (− 0.26 cm [caudally] vs. + 0.09 cm [cranially], *p* = 0.01), but similar shifts in the AP (− 0.13 cm vs. + 0.04 cm, *p* = 0.08) and RL direction (+ 0.00 cm vs. + 0.08 cm, *p* = 0.35), were found in patients with mean BHL > 1.4 L compared to those with BHL < 1.4 L, respectively (Fig. 5b). The correlations were not statistically significant between BHL and positional errors in AP (*r =* 0.269, *p* = 0.18), CC (*r =* 0.041, *p* = 0.84), and RL (*r =* 0.024, *p* = 0.91) directions, respectively. Other patient-related factors, including age, liver volume, and gross tumor volume, were not significantly associated with positional error (Table 2).

**Table 2:** Comparison in positional errors between subgroups

Subgroup	n	Average positional shift ± SD (mm)		
		AP	CC	RL
Overall	28	− 0.48 ± 2.54	− 0.85 ± 3.72	+ 0.41 ± 2.35
Age				
≤ 60 y/o	13	− 0.17 ± 2.71	−1.89 ± 2.95	+ 0.71 ± 2.45
> 60 y/o	15	−0.74 ± 2.35	+ 0.06 ± 4.07	+ 0.14 ± 2.23
*p value*		*0.58*	*0.17*	*0.54*
BMI				
≤ 22.9	14	−0.73 ± 2.73	− 0.19 ± 2.32	+ 0.66 ± 2.70
> 22.9	14	−0.22 ± 2.30	− 1.50 ± 4.63	+ 0.27 ± 1.91
*p value*		*0.61*	*0.38*	*0.59*
Mean BHL				
≤ 1.4 L	14	−1.34 ± 2.00	+ 0.90 ± 2.64	− 0.03 ± 2.30
> 1.4 L	14	+ 0.39 ± 2.72	−2.59 ± 3.82	+ 0.84 ± 2.32
*p value*		*0.08*	***0.01***	*0.35*
ΔBHL				
≤ 0.03 L	18	−0.15 ± 2.87	−0.81 ± 4.25	+ 0.66 ± 2.43
> 0.03 L	10	− 1.06 ± 1.65	−0.90 ± 2.50	−0.06 ± 2.14
*p value*		*0.31*	*0.95*	*0.45*
Liver volume				
≤ 1.3 L	13	−0.43 ± 1.59	−1.38 ± 3.80	+ 0.65 ± 1.92
> 1.3 L	15	−0.51 ± 3.14	−0.39 ± 3.59	+ 0.20 ± 2.65
*p value*		*0.93*	*0.50*	*0.62*
GTV				
≤ 60 cm^3^	14	−0.60 ± 2.06	− 1.49 ± 3.30	+ 1.09 ± 1.72
> 60 cm^3^	14	−0.35 ± 2.94	−0.20 ± 4.00	−0.28 ± 2.68
*p value*		*0.80*	*0.38*	*0.13*

Under the rigorous protocol for breath-hold precision, the positional errors did not significantly differ between patients with ΔBHL < 0.03 L and ΔBHL > 0.03 L in the AP (− 0.015 cm vs. -0.106 cm, *p* = 0.31), CC (− 0.081 cm vs. -0.090 cm, *p* = 0.95), and RL (0.066 cm vs. -0.006 cm, *p* = 0.45) directions (Table 2).

Height and weight correlated with mean BHL (*r =* 0.605 and 0.502, *p* = 0.001 and 0.007, respectively). The Pearson correlation coefficient between mean BHL and positional error in the CC direction was − 0.346 (*p* = 0.071). BHL was not correlated with positional errors in the AP (*r =* 0.270, *p* = 0.165) or RL (*r =* 0.244, *p* = 0.211) direction. There was no correlation between ΔBHL and positional errors in the AP (*r =* − 0.147, *p* = 0.456), CC (*r =* 0.031, *p* = 0.874), or RL (*r =* 0.024, *p* = 0.902) directions.

## Discussion

Treating liver cancer with radiotherapy remains a challenge because of the surrounding critical organs. Although vertebral bodies have been used as alignment positions for liver radiotherapy, especially in the AP and RL directions, one study found that the errors in CC direction and the irregular three-dimensional liver motion could not be detected by orthogonal images [17]. On the other hand, kV-CBCT, with its volumetric information, provides improved accuracy of radiotherapy through visualization of the liver and the surrounding soft tissue. Using CBCT inevitably costs more than using two-dimensional images [16, 19, 20]. Therefore, to align the liver using an implanted radiopaque marker close to the target lesion under orthogonal image guidance is now a common and acceptable method [21]. Our study consistently demonstrated that the correlation of the target positions under CBCT image guidance was significantly higher in all three dimensions on orthogonal images aligned with the stent/lipiodol than in those with vertebral body alignment.

Normal liver tissue is sensitive to radiation, and breathing inevitably affects the liver position through diaphragm movement. Therefore, respiratory control is needed to reduce treatment uncertainty and achieve accurate dose coverage. With breath hold using an active breathing coordinator (ABC) (Elekta Oncology Systems, Crawley, UK), the intra-fractional positional error and reproducibility in hepatobiliary radiotherapy were all less than those reported previously [9, 10, 22]. The mean intra- and inter-fraction positional errors in the CC direction were 1.9 mm and 6.6 mm, the root-mean-square errors were 2.1 mm and 5.2 mm, and the reproducibility were 2.3 mm and 4.3 mm, respectively [9]. Another study similarly found mean intra- and inter-fraction positional errors in the CC axis of 1.7 mm and 3.7 mm, and found reproducibility of 1.5 mm and 3.4 mm, respectively [10]. Of note, the SDX system is designed with individual BHL and limited breath-hold range (BHL ± 0.1 L). Our results in positional error and reproducibility were consistent with those found with the ABC system. However, there has not been any direct study on the comparison between the limited breath-hold range with the SDX system and the breathing threshold method with the ABC system. In terms of peak exploratory flow (PEF), Fleisch meter by use of pneumotachograph demonstrated a more accurate PEF measurement than Wright meter and turbine spirometer [23]. Whether the pneumotachograph spirometer of the SDX system is more sensitive and accurate than the turbine spirometer of the ABC system remains to be validated.

However, reproducibility of breath hold is important in order to reduce positional errors. Inter-fraction variations in breath-hold position could exceed 4 mm with a range of 1–8 mm, especially in the CC direction, even when using a pneumatic abdominal compression belt to reduce respiratory motion [24]. Therefore, our study investigated the association between breath-hold variation and positional error with the breathing-hold range limited by the SDX system. In contrast, our results showed that breath-hold variation was not significantly associated with positional errors, which means visually guided voluntary breath hold and the breath-hold range limitation of the SDX system can maintain both breath-hold consistency and patient body conformity.

The reference value of pulmonary function was related to body factors, such as height, weight, body mass index (BMI), and gender [25]. We found that patients with mean BHL > 1.4 L were significantly taller and heavier, and had larger positional errors in the CC direction. The physical size of patients may affect the inspiration depth and further affect the accuracy of the CC position. Notably, significance was not shown with BMI, probably because the exclusively Asian patients in this study had a smaller and narrower range of BMI than that of other populations [26]. Although our data showed that mean BHL was significantly associated with positional errors, the mean shifts were less than our PTV margin (0.5 cm) expanded from clinical target volume. Such positioning confidence under image guidance undoubtedly helps the radiotherapy dose coverage meet the clinical goals [11, 27].

Limitations of this study should be acknowledged. First, the limited number of patients was a shortcoming because we excluded several patients who did not have stents or embolized materials for image guidance. A relative smaller sample size might lead to bigger variation in statistical analysis of certain parameters. Further expansion of sample size could be overcome by continuing to enroll patients in the future.

Second, we used orthogonal images rather than CBCT in data analysis. The image acquisition by old CBCT system could not be completed in a single breath hold, so the reconstructed CBCT from multiple breath holds may increase the uncertainty of alignment. This shortcoming could be overcome by new CBCT system, which can nowadays be complete within one single breath hold, but no data is available for potential difference between CBCT images from single and multiple breath holds. With higher correlation between the shifts on CBCT with stent or embolized materials than with vertebral body on orthogonal images, kV-orthogonal images with stent/lipiodol were used as the reference. Our ongoing work involves collecting more data from patients to expand our analysis and confirm data consistency.

## Conclusion

In this study, patients treated with hepatobiliary radiotherapy using the SDX system for breath holds demonstrated effective and accurate tumor motion reduction. Actual BHL but not breath-hold precision (ΔBHL) was associated with positional errors under a predefined rigorous breath-hold protocol. Patients with larger body weight and height had significantly larger BHL and greater caudally positional error. The findings indicate that body-specific BHL plays a crucial role in positional error with breath-hold respiratory control.

## Supplementary information

**Additional file 1: Supplementary Figure 1.** (A) Training and preparation sessions of SDX system included (a) the determination of the inspiratory capacity and (b) the selection of the volumetric value. (c) The breath-hold level was defined as 85% of the inspiratory capacity and (d) the breath-hold range was restricted within 0.1 L. (B) During real treatment, patients were instructed by traffic light icon to distinguish (a) non-breath-hold phase (beam off) and (b) breath-hold phase (beam on).

## Data Availability

The data that support the findings of this study are available from the corresponding author upon reasonable request.

## Abbreviations

SDX: Spirometric motion management system
CBCT: Cone-beam computed tomography
BHL: Breath-hold level
ΔBHL: Breath-hold level variation
IGRT: Image-guided radiotherapy
SBRT: Stereotactic body radiotherapy
PTV: Planning target volume
DIBH: Deep inspiration breath-hold
SDX system: SpiroDynr’X system®
ABC: Active breathing coordinator
PEF: Peak exploratory flow
