# Supplementary material for: Impact of breath-hold level on positional error aligned by stent/Lipiodol in Hepatobiliary radiotherapy with breath-hold respiratory control
Source: BMC Cancer. 2020 Jul 1;20:613. doi: 10.1186/s12885-020-07082-y (PMC7328270; doi:10.1186/s12885-020-07082-y)
Supplement: Supplementary file 1 — Additional file 1: Supplementary Figure 1. (A) Training and preparation sessions of SDX system included (a) the determination of the inspiratory capacity and (b) the selection of the volumetric value. (c) The breath-hold level was defined as 85% of the inspiratory capacity and (d) the breath-hold range was restricted within 0.1 L. (B) During real treatment, patients were instructed by traffic light icon to distinguish (a) non-breath-hold phase (beam off) and (b) breath-hold phase (beam on). [file 12885_2020_7082_MOESM1_ESM.docx]

**Supplementary figure 1**

(A) Training and preparation sessions of SDX system included (a) the determination of the inspiratory capacity and (b) the selection of the volumetric value. (c) The breath-hold level was defined as 85% of the inspiratory capacity and (d) the breath-hold range was restricted within 0.1 L. (B) During real treatment, patients were instructed by traffic light icon to distinguish (a) non-breath-hold phase (beam off) and (b) breath-hold phase (beam on).

**
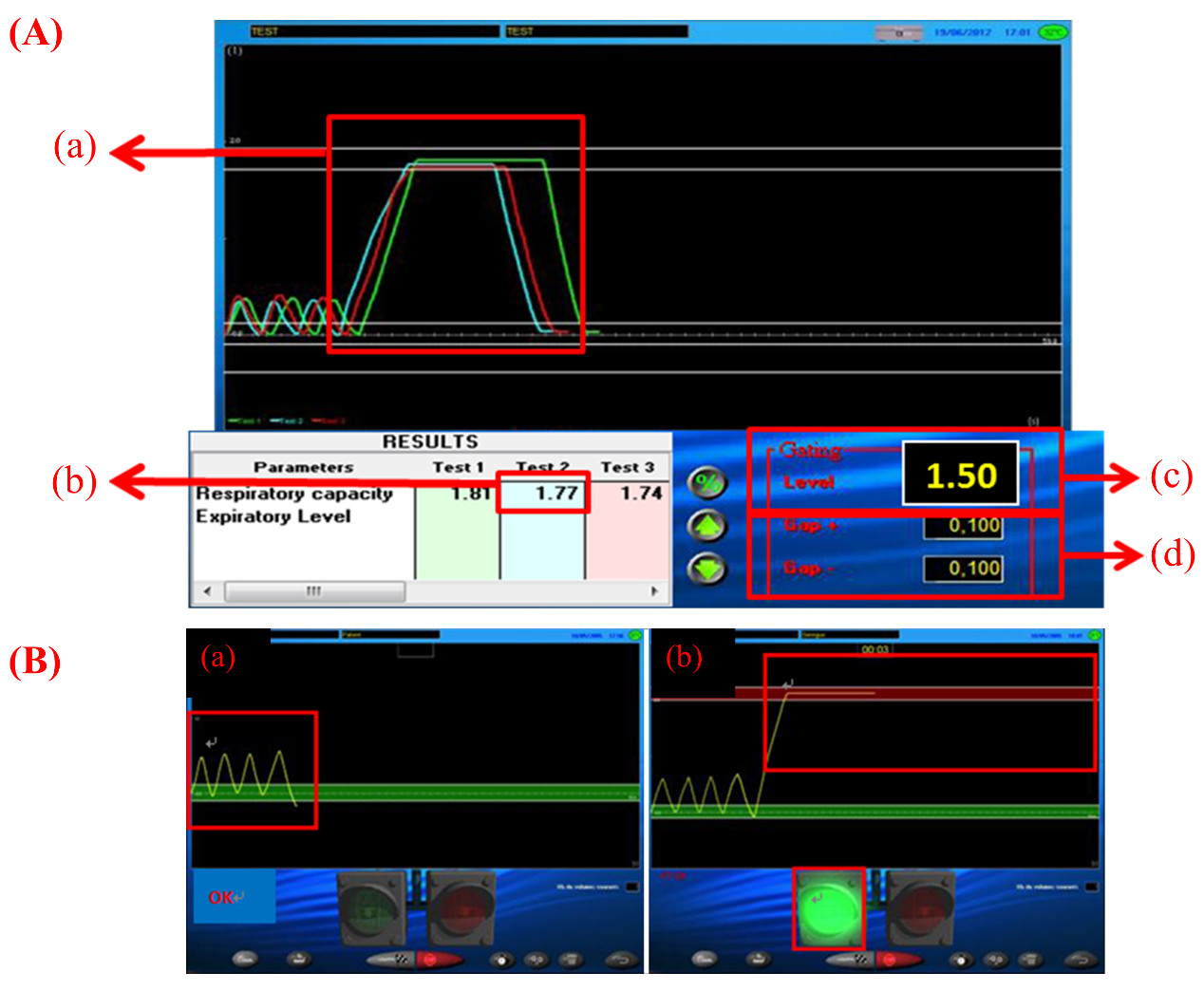
**
